# Inclusive study of peanut shells derived activated carbon as an adsorbent for removal of lead and methylene blue from water

**DOI:** 10.1038/s41598-024-63585-9

**Published:** 2024-06-12

**Authors:** Heba M. Hashem, Mahmoud El-Maghrabey, Rania El-Shaheny

**Affiliations:** Department of Pharmaceutical Analytical Chemistry, Faculty of Pharmacy, Mansoura University, Mansoura, 35516 Egypt

**Keywords:** Activated carbon, Water remediation, Adsorption, Peanut shells, Methylene blue, Lead, Pollution remediation, Chemistry

## Abstract

Green and efficient agro-waste-based activated carbon has been prepared utilizing peanut shells for adsorptive elimination of an industrial dye, methylene blue, and lead from polluted water. The carbonaceous biomass obtained from peanut shells was chemically activated using either NaOH, ZnCl_2_, or steam and characterized by scanning electron microscopy, Fourier-transform infrared spectroscopy, and N_2_ adsorption and desorption studies. The adsorption process was optimal for methylene blue at alkaline pH, while pH 4.5 was optimal for Pb (II) adsorption. The adsorption takes place through pseudo-second-order kinetic, and the rate-governing step of the adsorption procedure are intraparticle diffusion and film diffusion. Furthermore, the thermodynamics of the adsorption process has been studied, and the obtained Gibbs free energy (ΔG°) values are negative (− 35.90 to − 43.59 kJ mol^−1^) indicating the spontaneous adsorption of the investigated pollutants on the prepared activated carbon. As per the correlation coefficient, the obtained results were best fit by the Langmuir isotherm with maximum adsorption capacity of 303.03 mg g^−1^ for methylene blue and 130.89 mg g^−1^ for Pb (II). The activated carbon successfully removed methylene blue and Pb (II) with %removal exceeding 95%. The mechanisms of interaction of Pb (II) with the activated carbon is a combination of electrostatic interaction and ion exchange, while methylene blue interacts with the activated carbon via π–π interaction, hydrogen bonds, and electrostatic interaction. Thus, the prepared activated carbon has been employed to decontaminate wastewater and groundwater samples. The developed agro-waste-based activated carbon is a promising, cost-efficient, green, and accessible tool for water remediation.

## Introduction

Water is the essence of sustainability, as it is vital for socio-economic growth, healthy environments, and human life. United Nations-Water’s primary goal is *securing sustainable water for all,* which clearly focuses on the development aims of societies and fosters human goodness, leading to a healthy population, improved prosperity, and a protected environment. Climate change causes increasing water needs while declining water sources^1^. Nowadays, many regions in the world are experiencing increased demand for fresh water. It is a major challenge to get pure drinking water since the quality of fresh water is declining incessantly^2,3^. According to UNICEF, it’s predicted that half of the world’s residents will live in places suffering from water scarcity by 2025^4^. This challenges water executives to satisfy the requirements of growing societies, sensitive environments, farmers, energy manufacturers, and industry.

Pollution of surface and groundwater sources is another reason for the decline of freshwater sources, which is considered one of the most critical problems that can impact humans, animals, plants, and aquatic organisms. Before 2015, approximately 20% of global wastewater was well-treated. Meanwhile, about 70% of industrial wastewater is released without appropriate eradication in developing countries^4^. Aquifers are exhausted worldwide and are polluted due to numerous complications of saltwater intrusion, soil attrition, insufficient cleanliness, pollution of ground and surface waters by algae, chemicals, detergents, fertilizers, pesticides, heavy metals, etc. The classical approaches for the elimination of organic contaminants and heavy metals from wastewater incorporate chemical precipitation, membrane processes, ion exchange, photocatalysis, and distillation, which are costly and not suitable for the removal of all contaminants^5,6^.

In the same vein, the adsorption technique is considered the most widely used method for water purification due to its prominent merits, including cost-efficiency, feasible scalability, and a wide range of adsorbents such as magnetic nanoparticles^7^, clay minerals^8^, synthesized polymers^9–11^, or carbonaceous adsorbent^12,13^. These adsorbents are currently utilized for the elimination of various pollutants like dangerous heavy metals like iron, lead, and mercury, dyes such as crystal violet and rhodamine B, and organic pollutants such as pesticides, antibiotics, and phenol.

In this context, activated carbon (AC) prepared from agricultural by-products and wastes are excellent substitute adsorbents for the elimination of organic contaminants and heavy metals from waste and groundwaters since they are cheap, sustainable sources with high carbon, low ash, and rational hardness. AC is a treated carbon material with a porous assembly and a high interior surface area. AC contains mostly carbon (87–97%), in addition to some elements like hydrogen, oxygen, sulfur, and nitrogen. It can adsorb numerous matters from both gas and liquid media^2^. The means of preparation of AC include physical activation at temperatures 800–1000 ºC and chemical activation that integrates carbonization and activation in a single-step process at a relatively lower temperature. Chemical activation is mostly done using ZnCl_2_, H_3_PO_4_, H_2_SO_4_, KOH, or NaOH. These chemicals act as both oxidizing and dehydrating reagents, allowing simultaneous carbonization and activation. The important advantages of chemical activation are the possibility of activation at relatively low temperatures and the great yield^3^.

Dyes are widely employed in many industries, like textiles, paints, paper products, and cosmetics. The textile industry is a chief source of water pollution^14^. Therefore, dyes are among the most abundant contaminants in wastewater^15^. Industrial wastewater discharge can contaminate rivers and other water assets since they are poisonous, mutagenic, and carcinogenic^16^. Furthermore, they can decrease the photosynthesis of marine flora, causing damage to the marine biota^17^. Among various dyes, methylene blue ([7-(dimethylamino) phenothiazin-3-ylidene] dimethylazanium chloride, MB), which is a synthetic cationic thiazine dye of an amorphous nature, is one of the most common dyes that should be eliminated from wastewater^3^.

In the same vein, heavy metals are hazardous, non-degradable water impurities that are biomagnified in the food chains. Their presence is damaging since they are stored in the bodies of humans or other organisms, leading to long-term health hazards and disorders^18^. Being small molecules with complex structures, they are challenging to remove. The primary origins of these metals include human activities like mining, fertilization, electroplating, and the car industry^19^. Lead, Pb (II), is a very dangerous metal that is ranked second in the record of dangerous substances by The Agency for Toxic Substances and Disease Registry^20–22^. The sources of lead-polluted water include industrial and wastewater discharges, pesticides, paints, water pipes, and leachate from lead-acid batteries^20^.

The present study focuses on the utility of AC prepared from peanut shells as an economical and widely accessible plant waste for the elimination of lead and MB from water solutions as models for water contaminants. Peanuts are cultivated in developing countries; more than 80 million tons of peanut shells are annually left as agricultural waste without any commercial use^23^. The fiber contains cellulose 35.7%, lignin 30.2%, hemicellulose 18.7%, and ash 5.9%^24^. Peanut shells are usually used as a low-value energy source, either burned in the field or discarded, which is harmful to the environment. The processing and transformation of peanut shells to AC with high adsorption capacity would lessen problems of disposal and management while producing a value-added product for groundwater and wastewater treatment to expand the AC market. The valorization of agricultural and industrial waste gained great attention from researchers since it is a cheap, non-toxic, and sustainable feedstock^25–28^. This approach supports the circular economy practice by decreasing material consumption, reusing agro-waste, and recycling materials.

Thus, the aim of this study is to utilize peanut shells to produce cheap AC that is effective for water purification as tested by adsorptive removal of Pb (II) and MB. The AC has been fully characterized, and the factors influencing the adsorption process and the adsorption mechanisms have been explored. The adsorption data have been compared with different kinetic and isotherm models. The prepared AC is proven as a versatile adsorbent that can be used for the elimination of contaminants of different natures and chemical properties. This is an extra advantage of the prepared AC since few ACs in the literature have succeeded in doing so. Additionally, it was found that non-treated leafy vegeables itself can adsorb heavy metals on its surface^29^. Furthermore, in contrary to the developed approach, many reported literature on water decontamination or metal capturing via adsorption are depending on adsorbants from synthetic origin such as (3-(3-(methoxycarbonyl)benzylidene) hydrazinyl)benzoic acid and 2-nitroso-1-naphthol anchord on silica^30,31^, *N*,*N*–bis(salicylidene)1,2–bis(2–aminophenylthio)ethane (BSBAE) embedded silica^32^, citric acid/reluctant^33^, Mg/Al-layered double hydroxide-Sodium hexametaphosphate composite^34^. This impart a significant merit to the developed approach regarding environmental safety and greenness. Consequently, the prepared AC has been successfully utilized to decontaminate real samples from wastewater and groundwater. The developed approach is eco-friendly, cost-efficient, and suitable for limited-resources communities.

## Experimental

### Instrumentation

A spectrophotometer from Chrom Tech. Co., Ltd. (USA) was utilized for absorbance measurement. An atomic absorption spectrophotometer (JBT 932 EA SP) was used for the measurement of lead concentration. Measuring the BET surface area (SBET) of the prepared carbons was done via nitrogen adsorption at 77 K using Surface Area and Size Analyzer, NOVA 2000 Series, from Quantachrome (Germany). A scanning electron microscope from JEOL, Japan, was applied to examine the surface of the carbons. Fourier transform infrared spectroscopy (FT-IR) spectra were scanned using the Jasco instrument (Model 6100, Japan). A Hi 931401 HANNA pH meter (Portugal) was utilized.

### Materials and reagents

Peanut shells were purchased from Egyptian markets. Methylene blue, ZnCl_2_, NaOH, and HCl were obtained from Sigma-Aldrich. Lead nitrate and NaCl were supplied by BDH (UK). MB stock solution was prepared in distilled water.

### Synthesis of activated carbon from peanut shells

#### Method I

Peanut shells were finely ground and rinsed with HCl (0.5%) to eliminate all dust. The produced matrix was allowed to dry overnight in a furnace at 105 °C, then pulverized and sifted with a mesh (1–4 mm). The produced powder was transferred to a tabular furnace (600 °C) for 2 h to be carbonized. The obtained carbonized matrix (CM) was immersed in NaOH solution for 24 h in two immersion ratios (w/w): CM: NaOH of 1:1 (w/w) (C_Na_,1:1) and 1:3 (w/w) (C_Na_, 1:3). Afterwards, the two mixtures were transferred into an oven 105 °C) to eliminate moisture overnight. Then, the activation was carried out at 750 °C for 2 h and then left at room temperature to cool. The produced AC was constantly rinsed with distilled water till the pH of the filtrate became within the range of 7.0–8.0. This was followed by overnight drying at 105 °C.

#### Method II

The ZnCl_2_-AC samples were synthesized by impregnation of CM in ZnCl_2_ in two immersion ratios: CM: ZnCl_2_ of 2:1, w/w (C_Zn_, 2:1) and 1:2, w/w (C_Zn_,1:2). The two mixtures were dehydrated by transferring to an oven (105 °C) to dry overnight then activated at 600 °C for 2 h. The AC was repetitively rinsed with distilled water. When the filtrate becomes free from chloride, it is allowed to dry overnight at 105 °C.

#### Method III

Activation of the CM with the steam flow (150 mL min^−1^) at variable times to get burn-off of AC samples of 20% and 48% (C_St_, 20% and C_St_, 48%).

### MB Adsorption experiments

Adsorption experimentations were done by mixing the synthesized AC with 50 ml aqueous solution of methylene blue (MB) as a model pollutant in 250 mL flasks and transferring it to a temperature-controlled shaking water bath at a stable shaking rate of 125 rpm. The experiments were performed at varied pHs (2–12), different dosages of AC, and different starting concentrations of MB.

The quantities of MB eliminated by AC (qe) were estimated using Eq. (1), and the % removed (R %) was estimated by Eq. (2)^35^.

1 $$q_{e} = \frac{C0 - Ce}{m} \times \, V$$

2 $$\% R \, = \frac{C0 - Ce}{{Ce}} \times \, 100$$

where *q*_*e*_ = the adsorbed quantity of MB (mg g^−1^), *C*_*0*_ and *C*_*e*_ are the starting and equilibrium liquid-phase concentrations of MB (mg g^−1^), respectively, V is the volume of the solution (L), and *m* is the weight of the sorbent used (g).

### Lead adsorption experiments

Pb (II) adsorption by AC was also investigated by the addition of 0.05 g AC to 50 ml of Pb (II) solution (50 ppm) within the pH range (1.5–7.0). The mixtures were shaken at room temperature at 150 rpm for 24 h, and samples were withdrawn for determination of metal concentration using an atomic absorption spectrophotometer. Furthermore, varied adsorbent concentrations from 0.05 to 0.25 g/25 mL have been tested. The quantities of Pb (II) eliminated by AC (q_e_) and % removed (R %) were calculated by Eq. (1) and (2), respectively.

### Regeneration and reusability experiment of the prepared AC

All the regeneration studies were performed with 0.1 g AC (C_Zn21_, C_Na11_, and C_St20_) mixed with 50 ml solution of 50 ppm MB and shaken for two h to achieve equilibrium at room temperature. The AC samples with adsorbed MB were filtered, washed, dried at 80 °C, regenerated using 0.2 M NaCl, and shaken at 25 °C for 2 h for regeneration. Then, the regenerated ACs were again placed in the flask for adsorption according to the procedures described earlier, with the adsorption-–regeneration process occurring in four cycles.

## Results and discussion

### Description of AC prepared from peanut shells.

#### Surface area analysis of AC

The surface area and porous assembly of carbonaceous materials are significant aspects in controlling their adsorption competencies. The adsorption/desorption of N_2_ on the prepared AC was studied at 77 K (Fig. S1, supplementary material) and analyzed by the BET equation^36^. The isotherms for carbon C are basically type I as per the BDDT categorization, exhibiting no hysteresis loop. The isotherms of the prepared AC (C_St20_, C_St48_, C_Zn21_, C_Zn12_, C_Na11_, and C_Na13_) demonstrate mixed features of types I and IV being less steep with closed hysteresis loops^37^.

**Figure 1:**
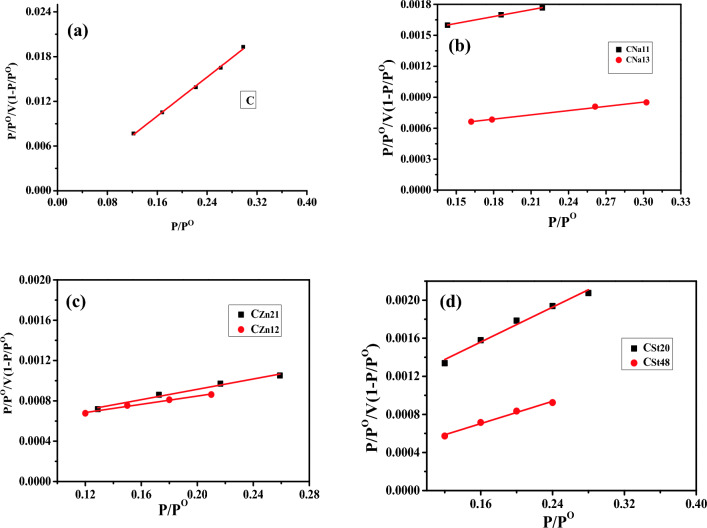
Surface area analysis of (**a**) non-activated carbon, (**b**) NaOH-activated carbons at ratios of 1:1 and 1:3 (Carbon: NaOH), (**c**) ZnCl_2_-activated carbons at ratios of 2:1 and 1:2 (Carbon: ZnCl_2_), and (**d**) steam-activated carbons (20% and 48% burn-off).

The surface area of the AC samples was measured from the linear BET plots of N_2_ adsorption at 77 K (Fig. 1). The surface area of non-activated carbon (non-AC) is very low relative to AC. This is attributed to the high degree of microporosity of AC^36^. The SBET of C_Na13_ and C_Zn12_ are significantly greater than C. Meanwhile, the NaOH-AC sample (C_Na13_) has the highest surface area value among all prepared AC. Furthermore, the activation of carbon with steam significantly increased the surface area. Furthermore, the total pore volume of non-activated samples is very low relative to AC. The pore radius ranged between 0.4 and 0.5 nm for the activated samples.

#### Surface pH and point of zero charge of AC

The pH at the point of zero charge (pH_pzc_) is the pH at which the surface functional groups do not contribute to the pH of the solution^38,39^. The pH_pzc_ of the slurries of C_St20_, C_St48_, C_Zn12_, C_Zn21_, C_Na11_, and C_Na13_ were found to be 8.20, 8.30, 6.57, 6.66, 8.77, and 8.90, respectively. This indicates that the base functional groups slightly predominate on the surface of C_Na11_, C_Na13_, C_St20,_ and C_St48_, while the pH of slurries of C_Zn12_ and C_Zn21_ were found to be neutral.

#### FTIR analysis of surface chemistry

**Figure 2:**
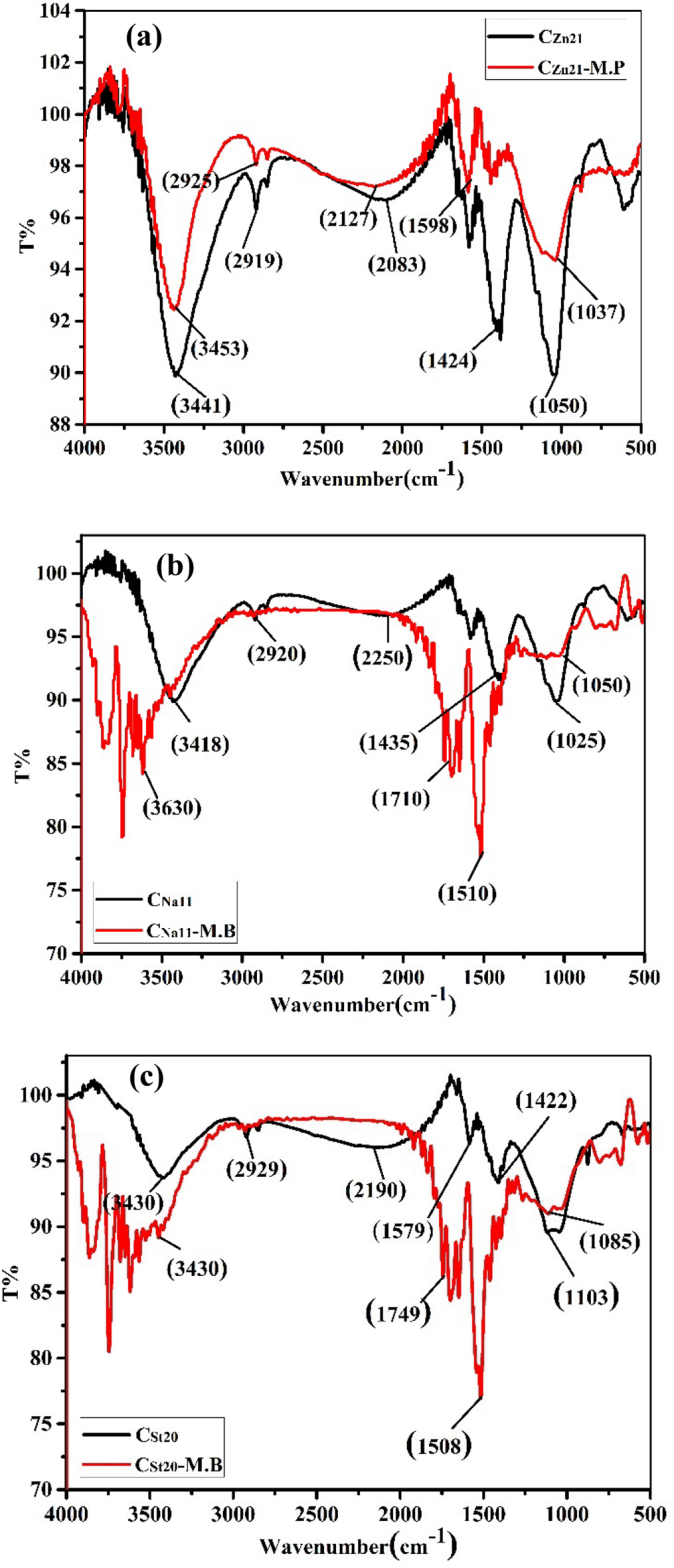
FTIR spectra of (**a**) C_Zn21_, (**b**) C_Na11_, and (**c**) C_St20_ before and after the adsorption of MB.

The spectra from FTIR analysis of the prepared AC have been examined (Fig. 2). The FTIR spectra of C_Zn21_, C_Na11_, and C_St20_ showed a broad band at 3414–3446 cm^−1^ allocated to O–H stretching. In addition, the peaks that appeared at 1034–1116 cm^−1^ are ascribed to the C–O stretching in alcoholic groups, and the bands detected at 610–875 cm^−1^ are ascribed to in and out plane ring deformation in benzene rings^40^. These findings suggest that the AC shows surface-OH groups.

#### Scanning electron microscopy (SEM) for morphological and pore structure characterization

**Figure 3:**
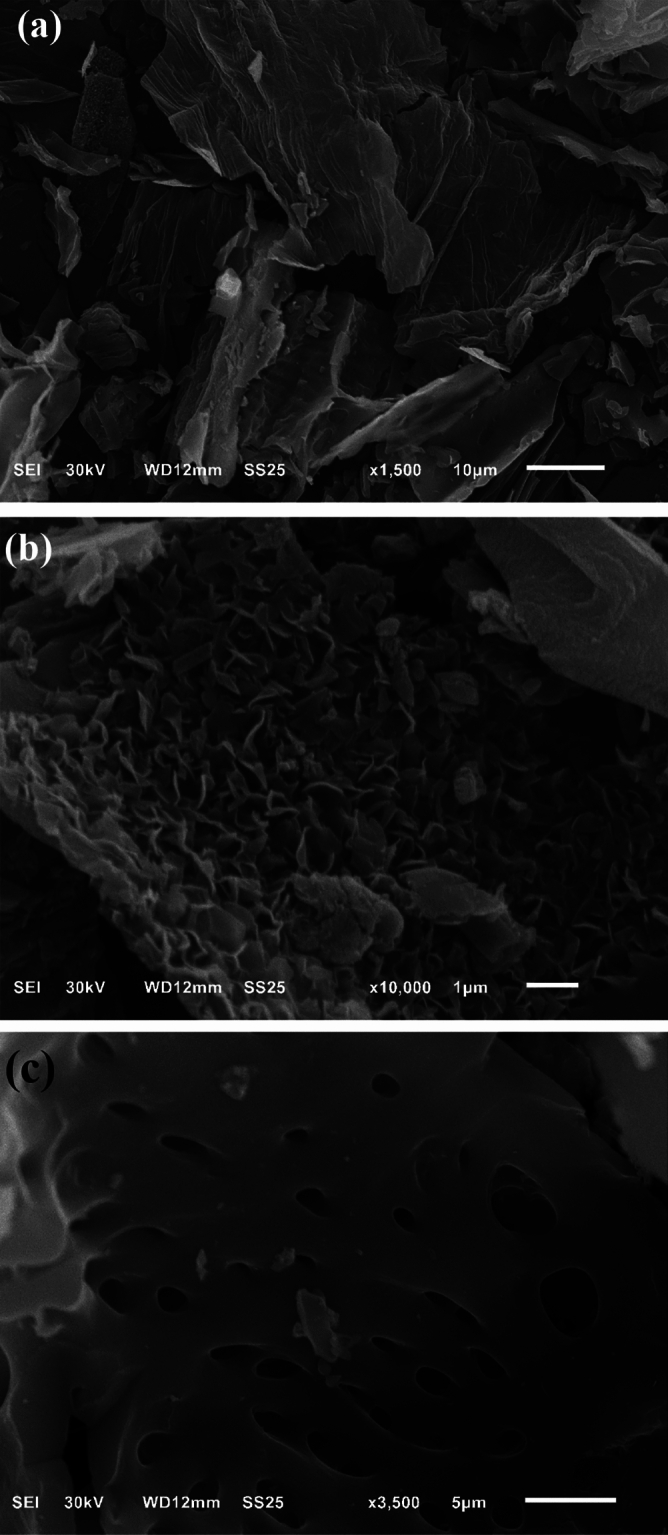
SEM images of (**a**) carbonaceous precursor, (**b**) activated carbon with NaOH, and (**c**) activated carbon with ZnCl_2_.

Scanning electron microscopy (SEM) offers data about the morphological features of the AC (Fig. 3). It is evident that activations result in significant surface alterations of the particles since the activation with NaOH or ZnCl_2_ leads to greater pores (dark areas) and lesser carbon matrix (pale gray area).

### Results of adsorption experiments of MB by the prepared AC

#### FTIR for AC after adsorption of MB

The FTIR spectra of AC exhibited the following significant alterations following the adsorption of MB (Fig. 2):

1. For C_Zn21_-MB: The manifestation of a band at 1424 cm^−1^ is referred to as C–H groups of alkanes (Fig. 2a).
2. For C_Na11_-MB: The bands at 3418, 2920, and 2250 cm^−1^, which are referred to as the O–H group, C–H group, and C–C stretching of aromatic moieties, respectively, disappeared, indicating the involvement of these groups in the C_Na11_-MB interaction. The manifestation of peaks at 1710 cm^−1^ and 1510 cm^−1^ (Fig. 2b) indicated the appearance of the C=N group and aromatic C═C of MB, respectively. This confirms the adsorption process.
3. For C_St20_-MB, the absence of the bands at 3430 cm^−1^, 2929 cm^−1^, and 2190 cm^−1^ implied the absence of O–H, C–H bonds, and C–C bonds, respectively. The new peaks that figured at 1749 and 1508 cm^−1^ (Fig. 2c) are allocated to the C=N group and aromatic C═C of MB, respectively.

#### Effect of initial pH on the adsorption of MB by AC

**Figure 4:**
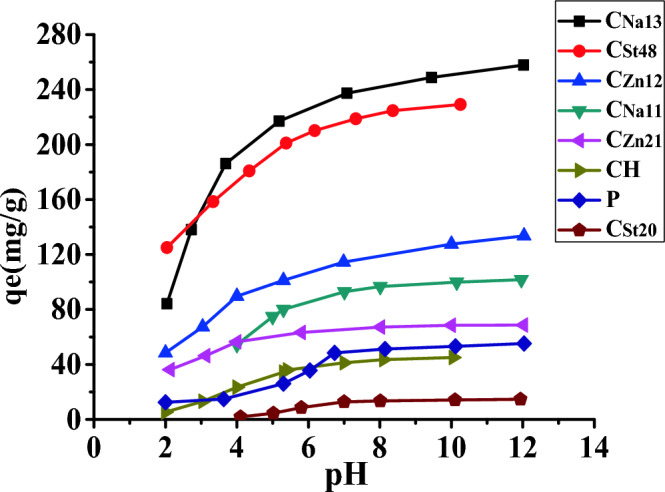
Effect of pH on the adsorption of MB on different AC.

Figure 4 demonstrates the impact of pH on the adsorption of MB on the AC. Increasing the original pH of the dye solution over the range of 2–12 leads to a stepwise increase of the adsorption (Fig. 4). The solution pH influences both the charge of the AC surface and the extent of dissociation of the adsorbates. Yet, the adsorption may also be affected by hydrogen bonds or π–π interaction^41^.

The weaker adsorption of MB at low pH is possibly attributed to the competing of H^+^ ions with the cationic site of the dye for adsorption spots of AC. Besides, at acidic pH, the –OH groups on the AC surface are non-dissociated; thus, electrostatic interaction between AC and MB is minimal. On the other hand, increasing the pH is associated with a corresponding increase in the quantity of MB adsorbed on the AC surface. This is attributed to the deprotonation of the surface –OH groups of AC and subsequent enhancement of the electrostatic interaction of the positively charged nitrogen of the cationic dye and the surface negative groups (–O^−^) of the AC.

#### The effect of the initial concentration of MB on the adsorption capacity of AC

**Figure 5:**
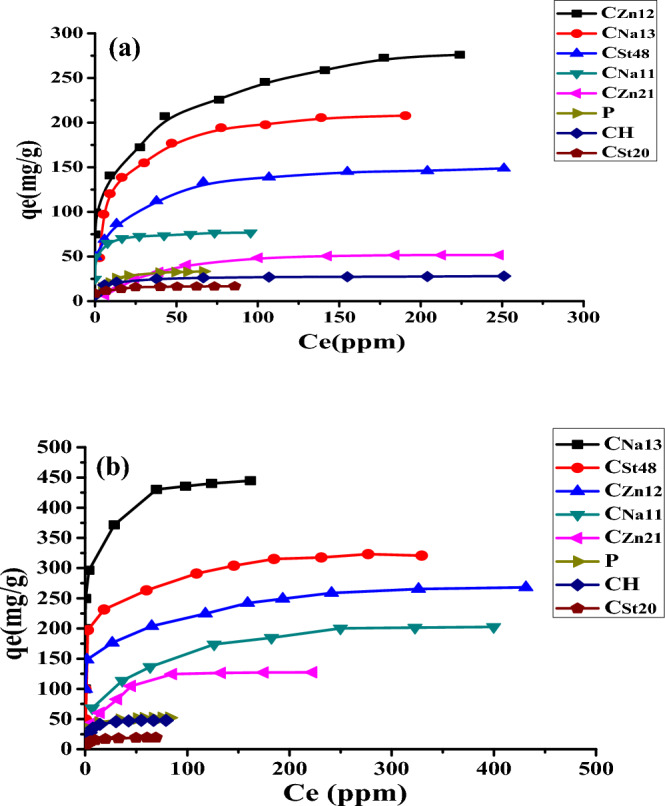
Effect of initial concentration of MB on the adsorption capacity of AC at (**a**) 28 and (**b**) 50 °C.

The equilibrium isotherm depicted in Fig. 5 showed that the adsorption capacity for AC improved by increasing the original MB concentration because of the rise in the key force of the concentration gradient by increasing MB's original concentration from 20 to 600 mg L^−1^. This reveals that the adsorption depends on the starting concentration of MB. At low concentrations, the ratio of (the initial number of MB dye molecules: the available surface area) is low. Thus, the adsorption did not depend on the original MB concentration. Yet, at high concentrations, the free adsorption sites are limited, and the adsorption % of MB dye is dependent upon its original concentration. Equilibrium has been established at two temperatures (28 and 50 °C) for all AC.

#### Effect of contact time of MB with AC

**Figure 6:**
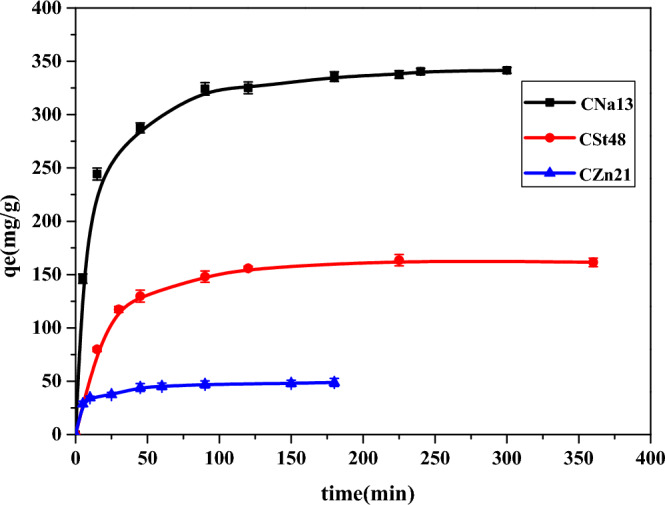
Effect of contact time on the adsorption of MB on C_Na13_, C_St48_, and C_Zn21_ samples.

A series of experiments have been performed to optimize the adsorption time at initial concentrations of 500 ppm by C_Na13_, C_St48,_ and C_Zn21_ samples for n = 3 (Fig. 6). The adsorption of MB by ACs increased with the increase of contact time. The contact time needed to achieve equilibrium was about 10–15 min for all AC samples; the capacity uptake of MB at equilibrium on the C_Na13_, C_St48,_ and C_Zn21_ are 350 ± 2.76, 150 ± 4.02, and 50 ± 3.71 mg g^−1^, respectively. This indicates the effectiveness of the ACs in removing MB from water.

#### Effect of adsorbent dosage

**Figure 7:**
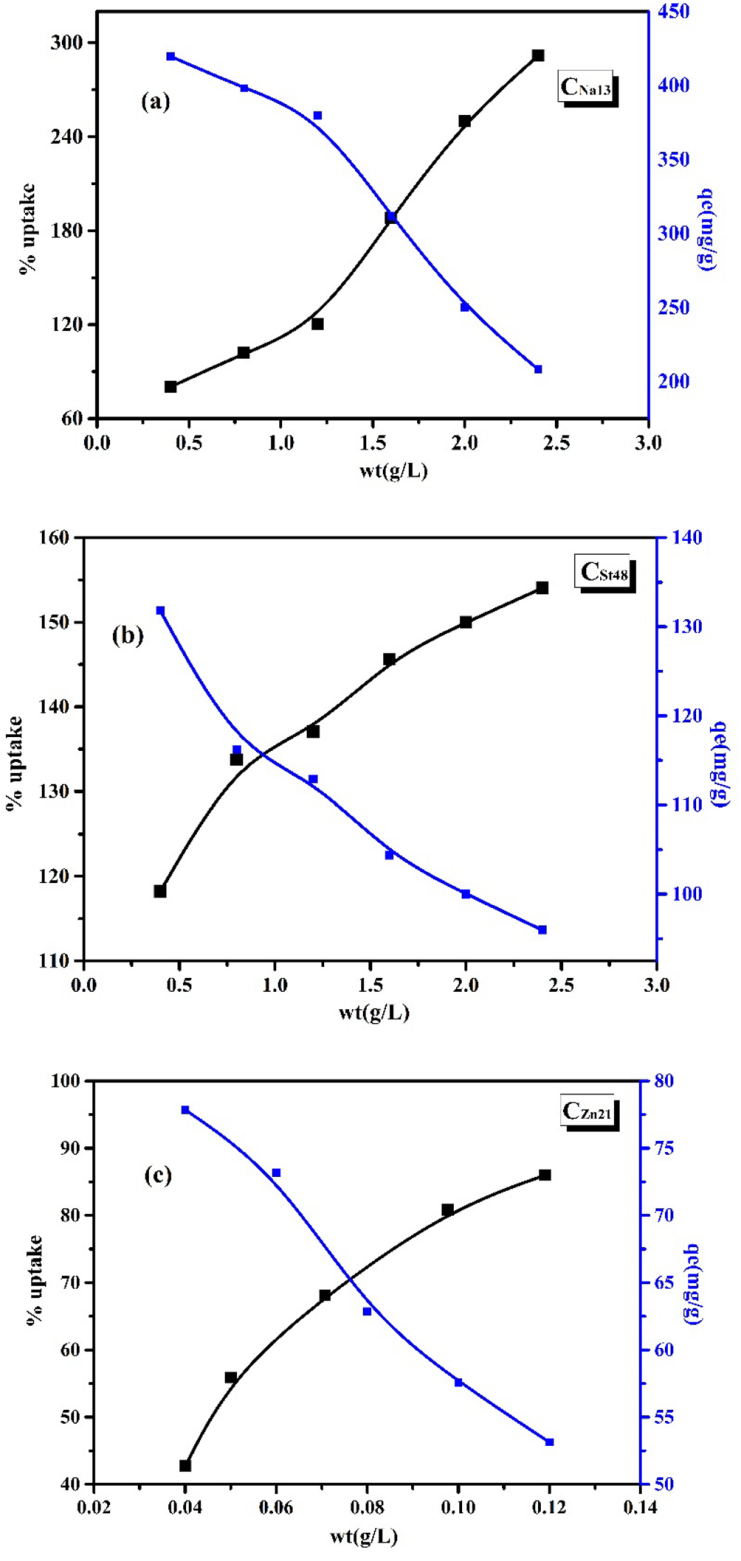
Effect of adsorbent dosage on the adsorption of MB on (**a**) C_Na13_, (**b**) C_St48_, and (**c**) C_Zn21_ samples.

The impact of AC dose on the adsorption of MB is presented in Fig. 7. Increasing the AC dose enhanced the adsorption % of MB due to the escalation in the accessible adsorption sites and surface area of AC.

#### Effect of foreign ions

Certain species commonly exist in natural water, like anions and cations, which may influence the adsorption of dyes on the AC. Therefore, the adsorption of MB by AC was assessed using some matrix ions such as F^−^, Cl^−^, Na^+^, K^+^, oxalate, and acetate. The inspection of the results (Table 1) shows that these ions had no negative influence on the adsorption of MB from water, where the %adsorption was in the range of 99.43–99.97%. This indicates the effectiveness of the AC for the eradication of MB from water, even in the presence of diverse foreign ions.

**Table 1:** Effect of different foreign ions on the % removal of MB (30 ppm) by C_Na13_, C_St48_, and C_Zn12_.

Foreign ions (200 ppm)	Adsorption % of MB		
	C_Zn12_	C_St48_	C_Na13_
F^−^	99.59	99.79	99.91
Cl^−^	99.49	99.64	99.94
Oxalate	99.60	99.80	99.92
Acetate	99.75	99.85	99.95
K^+^	99.43	99.65	99.94
Na^+^	99.59	99.62	99.97

#### Adsorption isotherm

**Figure 8:**
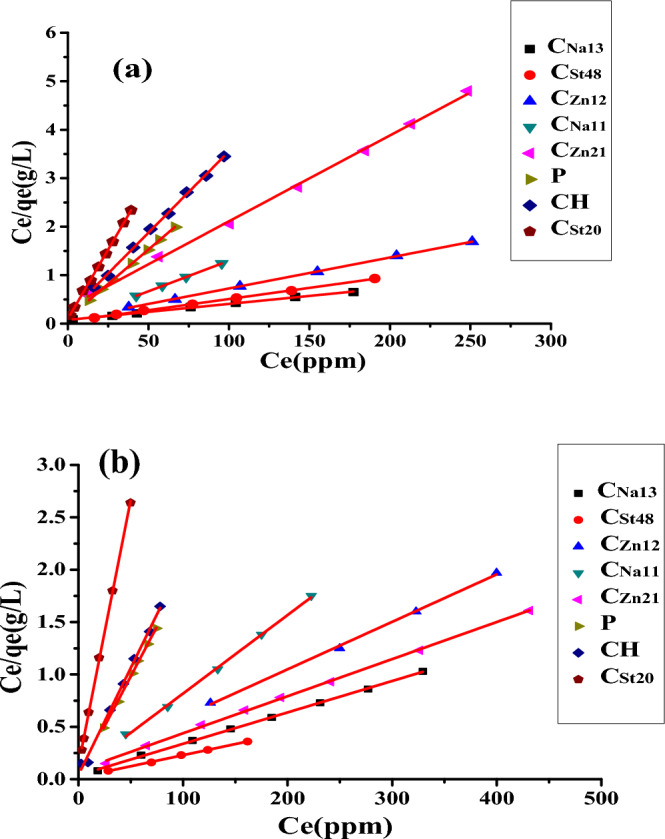
Langmuir plots at (**a**) 28 °C and (**b**) at 50 °C.

The adsorption equilibrium isotherm is significant in the investigation of adsorption systems. Different isotherms like Langmuir and Freundlich isotherms^42^ may describe the adsorption of AC. Langmuir and Freundlich isotherm models have been applied to explore the best fit to describe MB adsorption on AC. The linear form of Langmuir isotherm is shown in Eq. (3):

3 $$\frac{{{\varvec{C}}_{{\varvec{e}}} }}{{{\varvec{q}}_{{\varvec{e}}} }} = \frac{1}{{{\varvec{bq}}_{{\varvec{m}}} }} + \frac{1}{{{\varvec{q}}_{{\varvec{m}}} }}{\varvec{C}}_{{\varvec{e}}}$$

where b is the Langmuir equilibrium constant (L mg^−1^), and q_m_ (mg g^−1^) is the monolayer adsorption capacity. Both b and q_m_ are calculated from a curve of C_e_/q_e_ versus C_e_ (Fig. 8).

Langmuir isotherm is estimated by the separation factor, R_L_, which is outlined as follows:

4 $$R_{L} = \frac{1}{{1 + {\varvec{bC}}_{{\varvec{o}}} }}$$

where *C*_*o*_ is the original solute concentration.

The *R*_*L*_ value signifies the kind of isotherm and describes the adsorption process. The adsorption is considered unfavorable for R_L_ > 1, favorable if 0 < R_L_ < 1, or irreversible if R_L_ = 0^43^. In this investigation, the *R*_*L*_ values were within the range of (0–1) (Table 2). This proved that all AC exhibited favorable adsorption for MB. The favorability of the adsorption process followed the order of C_Na13_ > C_St48_ > C_Zn12_ > C_Na11_ > C_Zn21_ > P > CH > C_St20_ according to the calculated R_L_ values.

**Table 2:** Langmuir and Freundlich isotherms constants for adsorption of MB on AC at 28 and 50 °C.

AC aample	T = 28 ^o^C						T = 50 ^o^C					
	Langmuir constants			Freundlich constants			Langmuir constants		Freundlich constants			
	q_m_(mg g^−1^)	b (L mg^−1^)	R²	K	n	R²	q_m_ (mg g^−1^)	b (L mg^−1^)	R²	K	n	R²
P	35.71	0.248	0.999	7.71	2.21	0.996	52.63	0.633	0.998	35.52	11.11	0.993
CH	29.41	0.173	0.999	13.07	5.59	0.995	50.00	0.488	0.997	16.45	2.87	0.993
C_St20_	18.18	0.423	0.997	8.09	4.22	0.989	19.61	0.575	0.999	10.70	6.80	0.969
C_St48_	217.39	0.069	0.999	47.00	4.26	0.994	333.33	0.098	1.00	38.09	3.27	0.996
C_Zn21_	56.50	0.053	0.999	1.59	1.22	0.997	120.27	0.080	0.996	82.27	4.70	0.992
C_Zn12_	166.67	0.042	0.997	78.89	4.03	0.996	248.33	0.061	0.999	174.16	9.17	0.977
C_Na11_	79.37	0.315	0.999	58.56	15.63	0.994	139.87	0.328	0.999	53.52	6.71	0.982
C_Na13_	303.03	0.096	0.999	65.37	4.00	0.990	500.00	0.119	0.999	257.24	8.77	0.983

**Figure 9:**
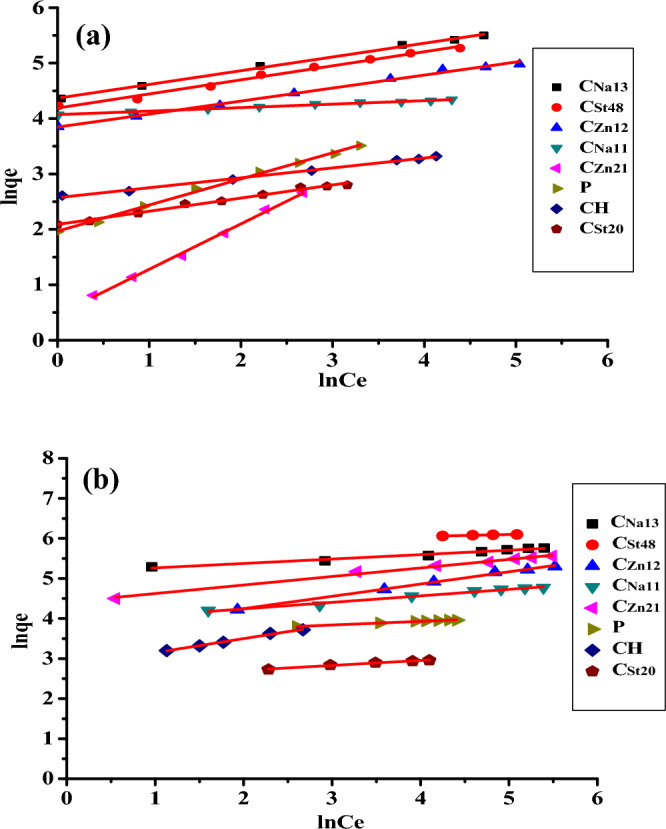
Freundlich plots at (**a**) 28 °C and (**b**) 50 °C.

Freundlich linear isotherm^44^ equation is shown below:

5 $${\mathbf{Ln}} \, {\mathbf{q}}_{{\mathbf{e}}} = \, {\mathbf{Ln}} \, {\mathbf{K}}_{{\mathbf{f}}} + \frac{1}{{\varvec{n}}}\user2{ }{\mathbf{lnC}}_{{\mathbf{e}}}$$

where K_f_ (L g^−1^) is the Freundlich constant, and n is the Freundlich exponent. These factors are calculated from a graph of log q_e_ against log C_e_ (Fig. 9).

Table 2 shows the parameters for Langmuir’s and Freundlich isotherms at 28 and 50 °C. Inspection of the obtained results depicts:

1. Both Freundlich and Langmuir models are appropriate to express the adsorption of MB on the AC as indicated by the high values of correlation coefficient (R^2^) that lie in the range of (0.977–0.999). The model that better fits the adsorption of MB on all ACs is the Langmuir adsorption model, as revealed by the higher R^2^.
2. Increasing the temperature is associated with a corresponding increase in the adsorption capacity, indicating the endothermic character of MB adsorption on the AC.
3. The increase of the activating agent, ZnCl_2_ or NaOH, leads to an improvement in the adsorption ability of the AC.

The Dubinin–Radushkevich (D-R) isotherm model has been applied to assess the adsorption from an energy perspective and assumes that it occurs on the porous surface^45^. The D-R model states that the pore structure of the surface where adsorption occurs affects the adsorption potential. For small pores with extremely high adsorption power, the adsorption potential is considerable. As a result, the D-R isotherm indicates that the pores are filled in order of smallest to largest. The energy value derived from the D-R isotherm model provides details on the adsorption mechanism. Which chemical or physical adsorption mechanism is most successful throughout the adsorption process is indicated by the E (kJ/mol) value. If the magnitude of the E value is between 8 and 16 kJ mol^−1^, it refers to the chemical adsorption mechanism. On the other hand, if it is smaller than 8.0 kJ mol^−1^, the physical adsorption mechanism exhibits. For this study, all AC samples exhibit low free energy values of less than 8.0 kJ mol^−1^, indicating that the adsorption of MB is mostly a physisorption process. The mean free energy E of adsorption per molecule of the adsorbate when it is transferred from the solution to the solid surface is proportional to the constant β, as shown in Table 3.

**Table 3:** Results for Dubinin-Radushkevich (D-R) isotherm for adsorption of MB on ACs at 28 and 50 °C.

AC sample	T = 28 °C				T = 50 °C			
	β (mol^2^ kJ^−2^)	E (kJ mol^−1^)	q_m_ (mg g^−1^)	R^2^	β (mol^2^ kJ^−2^)	E (kJ mol^−1^)	q_m_ (mg g^−1^)	R^2^
P	4.51 × 10^–6^	0.333	34.122	0.879	1.80 × 10^–6^	0.527	50.217	0.848
CH	2.13 × 10^–6^	0.485	27.316	0.782	1.81 × 10^–6^	0.527	50.200	0.866
C_St20_	2.27 × 10^–6^	0.469	15.964	0.997	2.99 × 10^–6^	0.408	20.096	0.993
C_St48_	4.87 × 10^–6^	0.320	130.221	0.740	6.43 × 10^–7^	0.881	287.490	0.570
C_Zn21_	1.93 × 10^–5^	0.161	45.777	0.939	1.36 × 10^–6^	0.605	106.514	0.479
C_Zn12_	1.44 × 10^–6^	0.589	223.158	0.794	1.13 × 10^–6^	0.664	235.179	0.720
C_Na11_	9.07 × 10^–7^	0.742	75.476	0.708	8.14 × 10^–6^	0.247	172.844	0.706
C_Na13_	3.32 × 10^–6^	0.388	179.201	0.908	9.51 × 10^–7^	0.725	420.179	0.872

#### Adsorption kinetics modeling

Kinetic modeling of adsorption data has been evaluated to validate the application of the adsorptive removal model in real industries^46^. Pseudo-first-order and pseudo-second-order models have been studied to explain the kinetics of the adsorption of MB on AC.

**Figure 10:**
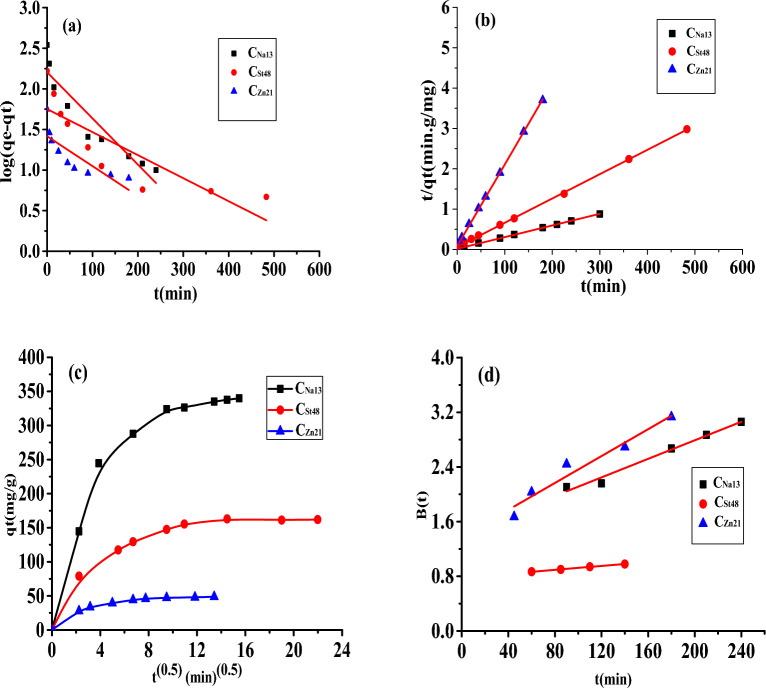
Investigation of the adsorption of MB on AC by (**a**) pseudo-first-order kinetic model, (**b**) pseudo-second-order kinetic model, (**c**) intraparticle diffusion, and (**d**) Boyd model.

The pseudo-first-order kinetic model is stated by Eq. (6):

6 $${\mathbf{log}}\left( {{\mathbf{q}}_{{\mathbf{e}}} - {\mathbf{q}}_{{\mathbf{t}}} } \right) = {\mathbf{logq}}_{{\mathbf{e}}} - \frac{{{\varvec{k}}_{1} }}{2.303}{\mathbf{t}}$$

where q_e_ and q_t_ (mg g^−1^) are the quantities of MB adsorbed at equilibrium and at time t, respectively. k_1_ is the equilibrium constant (min^−1^) calculated from the slope of the plot of Ln (q_e_−q_t_) against t in Fig. 10a.

The pseudo-second-order kinetic model is expressed by Eq. (7):

7 $$\frac{{\varvec{t}}}{{{\varvec{q}}_{{\varvec{t}}} }} = \frac{1}{{{\varvec{k}}_{2} {\varvec{q}}_{{\varvec{e}}}^{2} }} + \frac{1}{{{\varvec{q}}_{{\varvec{e}}} }}{\varvec{t}}$$

where K_2_ (g mg^−1^ min) is the equilibrium rate constant for the pseudo-second-order adsorption, and q_e_ can be calculated from the plot of t/q_t_ against t (Fig. 10b)^47^.

The obtained results are demonstrated in Table 4. For all investigated AC (C_Na13_, C_St48_, and C_Zn21_), the correlation coefficient R^2^ of pseudo-second-order plots was very close to 1 and much greater than R^2^ of the pseudo-first-order plots. Furthermore, the hypothetical q_2_e value calculated from the pseudo-second-order plot was very approximate to the experimental value of q_exp_ (Table 4), while the hypothetical q_1_e value assessed from the pseudo-first-order kinetic gave values very different from the experimental values (q_exp_). This indicates that the pseudo-second-order model is the applicable model to explain MB adsorption on the AC.

**Table 4:** Kinetic parameters for MB adsorption applying different kinetic models.

Kinetic model	Parameter	C_Na13_	C_St48_	C_Zn21_
	qe.exp (mg g^−1^)	350.00	150.00	50.00
First-order kinetic Eqs.	q_1_e (mg g^−1^)	162.18	56.23	26.30
	K_1_ (min)^−1^ .10^3^	14.00	6.90	9.20
	R_1_^2^	0.864	0.688	0.617
Second-order kinetic Eqs.	q_2_e (mg g^−1^)	344.00	163.93	52.00
	K_2_ [g (mg min)^−1^].10^4^	5.12	7.44	20.00
	R_2_^2^	0.999	0.999	0.999
Intraparticle diffusion Eqs.	K_int_[mg (g min^1/2^)^−1^]	12.22	3.66	1.73
	C	175.43	98.04	28.92
	R^2^_int_	0.749	0.660	0.789
Boyd Eqs.	I	1.43	0.78	1.38
	R^2^	0.978	0.998	0.935

#### Investigation of the rate-limiting step for MB adsorption on AC

In most adsorption systems, the surface step is relatively fast, and the rate-controlling step is diffusion, either film or intraparticle diffusion. Thus, for the determination of the adsorption rate controlling step, the intraparticle diffusion and Boyd models were investigated.

The intraparticle diffusion model^48^ was tested by applying Eq. (8):

8 $$q_{t} = k_{int} t^{0.5} + \, C$$

Where *k*_*int*_ (mg g^−1^ min^−1/2^) is the adsorption constant, and *C* is the intercept of a plot of *q*_*t*_* against t*^*1/2*^ (Fig. 10c)^48^.

The results were also inspected by the Boyd model (Eq. 9)^48^ to investigate whether the adsorption takes place via an external diffusion or intraparticle diffusion.

9 $${\mathbf{F}}\left( {\mathbf{t}} \right) = 1 - \frac{6}{{{{\varvec{\uppi}}}^{2} }}\mathop \sum \limits_{{{\mathbf{n}} = 1}}^{\infty } \frac{1}{{{\mathbf{n}}^{2} }}\exp \left( { - {\mathbf{n}}^{2} {\mathbf{Bt}}} \right)$$

where *F* is the fractional equilibrium at time *t* and is obtained by Eq. (10).

10 $$F \, = \frac{{{\varvec{q}}_{{\varvec{t}}} }}{{{\varvec{q}}_{{\varvec{e}}} }}$$

B (t) is the mathematical function of *F,* and *n* is the infinite series solution.

The results obtained (Fig. 10c) show that the graph of *q*_*t*_ versus *t*^*0.5*^ of C_Na13_ for MB has two linear parts referring to two phases that come about throughout the adsorption. Furthermore, since the linear part of the line did not cross the coordinate origin, it is anticipated that two steps will govern the entire adsorption process. The first sharp linear segment is referred to as fast film diffusion, which involves the transfer of MB molecules from the solution to the AC exterior surface via diffusion across the boundary layer. The second linear segment of the plot represents slower MB molecule diffusion from the outer surface of AC into the pores^35,49^.

Boyd kinetic model (Fig. 10d) was also applied, and the calculated results demonstrated that the linear graph of B (t) versus (t) of C_Na13_ for MB doesn’t cross the coordinate origin, signifying that the process of adsorption is governed by film diffusion^48^.

Thus, it is concluded that both film and intraparticle diffusion are the rate-determining steps for the adsorption process. The sorbates move from the bulk solution to the boundary layer on the AC surface. This step is followed by film diffusion, where the sorbates cross the boundary layer, reach the adsorption sites on the AC surface, and interact via molecular interactions. Furthermore, sorbates diffuse into the pores of the adsorbent, including the mesopores and micropores^50^.

#### Thermodynamic studies

The impact of temperature on MB adsorption by AC has been investigated at 298 and 320 K. The thermodynamic factors, comprising Gibbs free energy change (ΔG^°^, kJ mol^−1^), enthalpy (ΔH^°^, kJ mol^−1^), and entropy (ΔS^°^, kJ (mol·K)^−1^) were estimated^51^.

The free energy change (ΔG^o^) of the adsorption was calculated from Eq. (11), standard enthalpy change (ΔH^o^) was calculated from Eq. (13), and standard entropy change (ΔS^o^) was also calculated from Eq. (14)^52,53^.

11 $${{\varvec{\Delta}}}G^{o} = - {\mathbf{RTlnK}}_{{\mathbf{c}}}$$

Here, the ideal gas constant, R is 8.314 J mol^−1^ K^−1^. T and Kc are the temperature in K, and the thermodynamic equilibrium constant, respectively. Kc was measured using Eq. (12)

12 $${\mathbf{Kc}} \, = \, {\mathbf{M}}_{{\mathbf{W}}} \times {\mathbf{55}}.{\mathbf{5}} \times {\mathbf{1000}} \times {\mathbf{b}}$$

where, Mw is the methylene blue molecular weight (g mol^−1^), the number of moles of water per liter is 55.5, and the Langmuir constant, b (L mg^−1^).

13 $${\mathbf{\Delta H}}^{{\mathbf{O}}} = {\mathbf{R}}\left( {\frac{{{\mathbf{T}}_{1} {\mathbf{T}}_{2} }}{{{\mathbf{T}}_{2} - {\mathbf{T}}_{1} }}} \right)*2.303{\mathbf{log}}\left( {\frac{{{\mathbf{b}}_{2} }}{{{\mathbf{b}}_{1} }}} \right)$$

14 $${\mathbf{\Delta S}}^{{\mathbf{O}}} = \frac{{{\mathbf{\Delta H}}^{{\mathbf{O}}} - {\mathbf{\Delta G}}^{{\mathbf{O}}} }}{{\mathbf{T}}}$$

Table 5 demonstrates the calculated values for thermodynamic factors for the adsorption of MB by the AC. The positive ΔH° value signifies the endothermic character of MB adsorption by the AC. This explains the favorable adsorption of MB by the AC at elevated temperatures. In addition, the positive ΔS° proposes the augmented randomness at the solid/solution boundary throughout MB adsorption. The negative ΔG° implies the spontaneity of the adsorption. The value of Δ*G*^°^ improved at higher temperatures suggesting enhanced adsorption^35^.

**Table 5:** Thermodynamic parameters for the adsorption of MB on AC.

Sample	∆G°(kJ mol^−1^)		∆H° (kJ mol^−1^)	∆S° (kJ mol^−1^ K^−1^)
	T = 301 K	T = 323 K		
P	− 38.28	− 43.59	34.43	0.241
CH	− 37.38	− 42.89	34.43	0.238
C_St20_	− 39.61	− 43.33	4.43	0.146
C_St48_	− 35.08	− 38.58	12.89	0.159
C_Zn21_	− 34.42	− 38.04	15.13	0.164
C_Zn12_	− 33.83	− 37.31	13.71	0.157
C_Na11_	− 38.88	− 41.83	0.83	0.131
C_Na13_	− 35.90	− 39.10	7.89	0.145

#### Mechanism of MB Adsorption by the prepared AC

**Figure 11:**
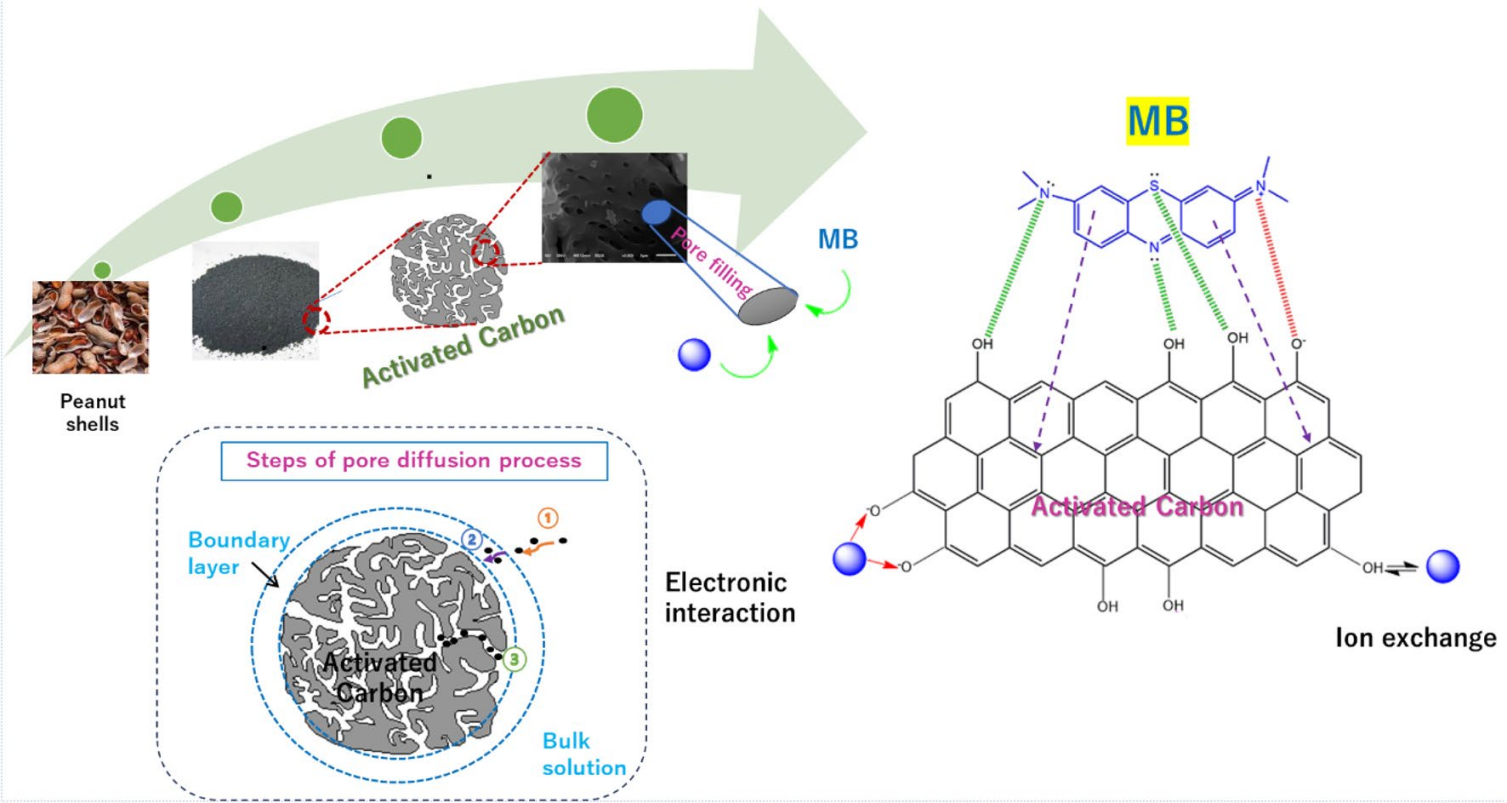
Diagram demonstrating the synthesis route of AC, possible MB adsorption mechanisms (green dots denote H-bonding, red dots denote electrostatic interaction, and purple dotted arrow denotes π–π interaction), and possible Pb (II) adsorption mechanisms (the blue ball represents Pb(II)) by the agro-based AC. The inset shows the steps of pore diffusion.

The adsorption of cationic dyes like MB onto AC is probably affected by diverse interaction forces like the π–π interaction, hydrogen bonds, or electrostatic interaction. The π-π interaction involves the aromatic moiety in MB and the aromatic core of the AC. Furthermore, H-bonding between electronegative atoms in the dye molecules (–N– and –S–) and H-bond acceptor in the AC (*e.g.* OH). Besides, electrostatic interaction takes place between the positive nitrogen of imine group in MB and the negative O^−^ of AC. Additionally, pore filling is mainly a key mechanism in the adsorption process due to the presence of mesopores and micropores in AC, enhancing their adsorption abilities for large adsorbate molecules like dye molecules^54^. Figure 11 illustrates the foremost ways for the adsorption of MB by the AC.

### Adsorption experiments of lead (II) by the prepared AC

#### FTIR and SEM for AC before/after adsorption of lead (II)

**Figure 12:**
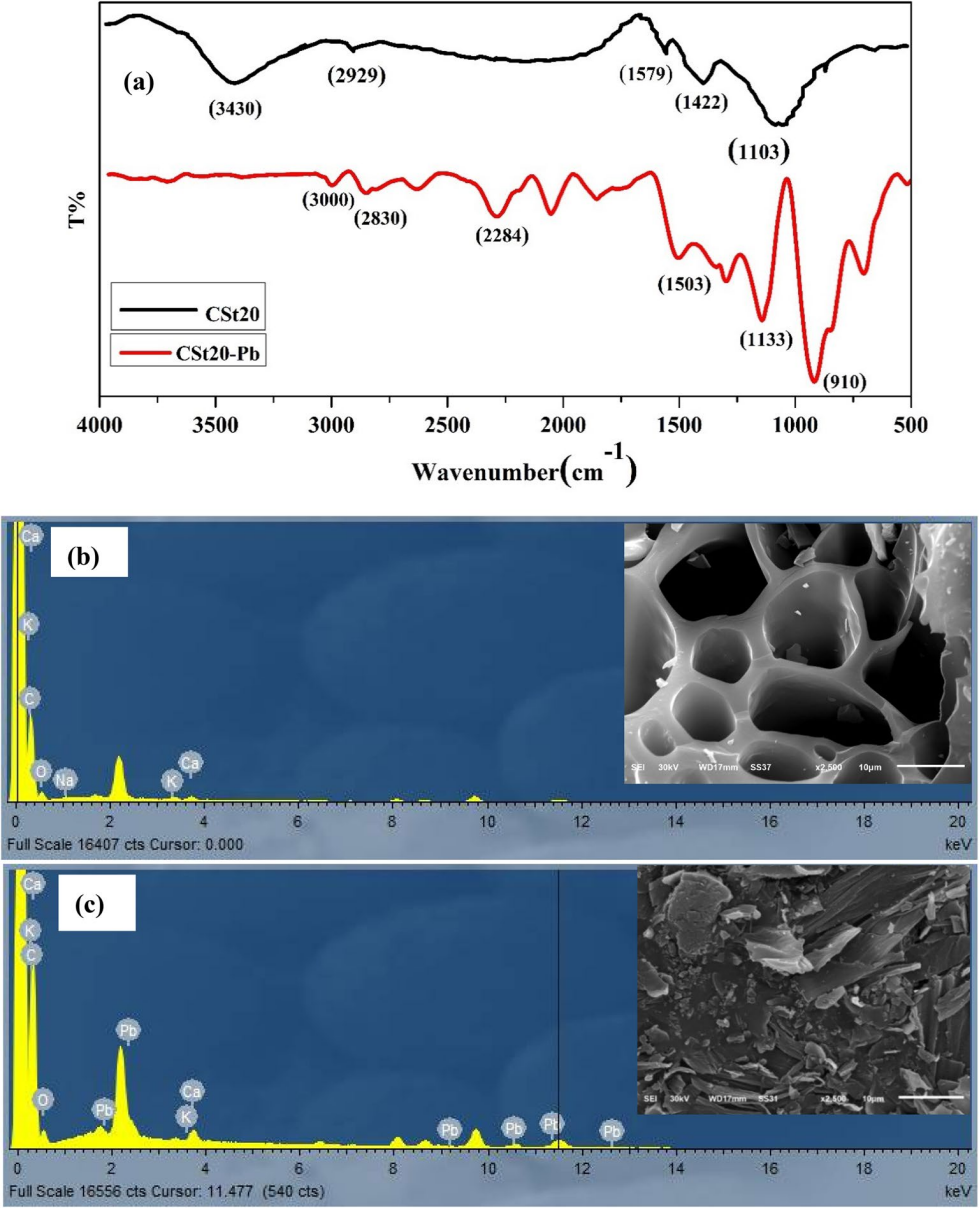
(**a**) FTIR spectra for activated carbon C_st20_ before and after adsorption of Pb (II). Scanning electron microscopy images (SEM) and Elemental analysis (EDX) of AC (**b**) before and (**c**) after adsorption of Pb (II).

The FTIR spectra of C_St20_ have been recorded before and after the adsorption of Pb (II) since the appearance/disappearance of peaks and shift of bands can be used as evidence for adsorption and to clarify the functional groups involved in the adsorption process. Observing Fig. 12a, one can see that the adsorption of Pb (II) on the surface of the AC leads to a change in positions and intensities of some peaks. Some changes in the wavenumber and intensities of the peaks are detected in the region from 500 to 1750 cm^−1^. Furthermore, the disappearance of the band at 3430 cm^−1^ suggests the involvement of the OH group of AC in its interaction with Pb (II) and the formation of a complex. In addition, a comparison of the SEM and EDX results obtained before and after Pb (II) adsorption by C_St20_ (Fig. 12b and 5c) corroborates such results. The presence of a peak in the EDX spectrum belonging to lead clearly proves the accumulation of lead (II) ions onto C_St20._ Figure 12a and b illustrate the SEM images of C_St20_ before and after adsorption of Pb (II), respectively. It is obvious that the surface texture of C_St20_ was entirely altered following the adsorption of Pb (II). The surface of the Pb (II)-loaded C_St20_ demonstrated that the surface of the C_St20_ was covered with Pb (II), and the pores were filled with the metal ions after the adsorption of Pb (II). This observation indicates that lead ions are bonded to the functional groups within the pores^55^.

#### Effect of initial pH

**Figure 13:**
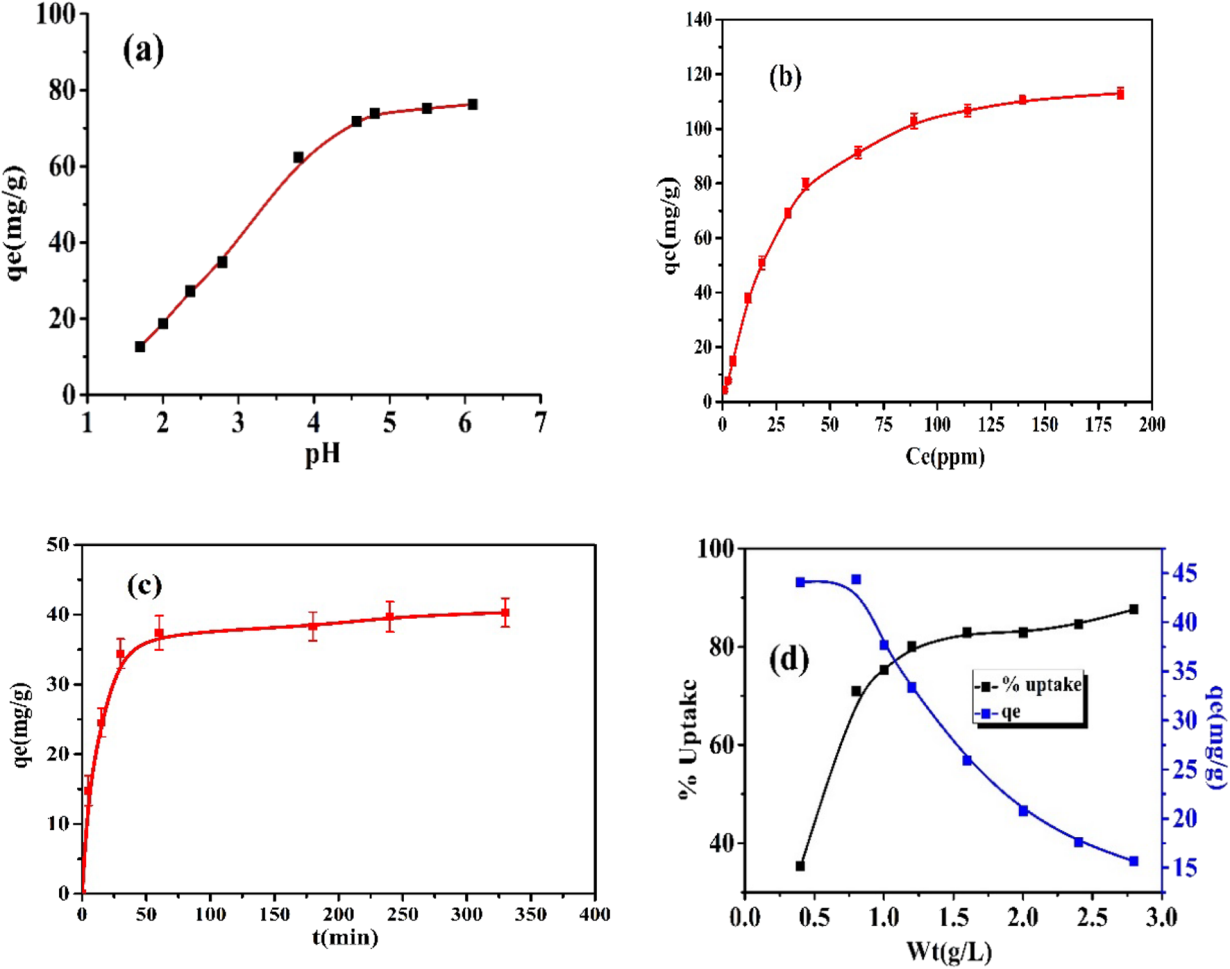
Effect of (**a**) pH, (**b**) initial Pb(II) concentration, (**c**) contact time, and (**d**) adsorbent dose on adsorption of Pb(II) on C_st20_ sample.

pH affects both the chemistry of AC and the speciation of lead. According to the literature, at pH between 1 and 5, the predominant species of lead is Pb (II), the predominant species is Pb (II) and Pb(OH)_2_ at pH 6, while Pb(OH)^3−^ and Pb(OH)_4_^2−^ are formed at pH between 7 and 12^56^. The preliminary pH of the medium was varied within the range of 1.6–7 to prevent precipitation of lead hydroxide. It is noted that the adsorption rate rapidly improved by increasing the pH from 2.0 to pH 4.5, then the rate slowly increased, and the adsorption reached its maximum level (Fig. 13a). The increase of the solution pH leads to ionization of the surface functional groups of AC (*e.g.*, OH), and it becomes more negatively charged. This boosts the electrostatic attraction of Pb (II) with the AC surface and improves the adsorption. pH 4.5 was chosen as the best pH for this investigation. On the other hand, the weak adsorption of Pb (II) at acidic pH (2–3) is explained by the competition of Pb (II) with the H^+^ for the adsorption sites on the AC surface^57,58^.

#### Effect of initial Pb (II) concentrations

As demonstrated in Fig. 13b, the adsorption enhanced as the initial Pb (II) concentration increased. The increase of the metal ion’s original concentration boosts the dynamic force of the concentration gradient^59^. The number of replicates were 3, and the SD values range from (0.522 to 2.867).

#### Effect of contact time

The impact of contact time of Pb (II) and C_St20_ was studied by changing the time over the range of 5 min to 24 h for n = 3. From Fig. 13c, the adsorption of Pb (II) ions is fast in the first hour due to the accessibility of plentiful adsorption sites on the surface of AC. After 1.5 h, equilibrium was reached, and constant adsorption was observed.

#### Effect of adsorbent dosage

The results of the adsorption of Pb (II) with varied dosages of AC are exhibited in Fig. 13d. Increasing the AC dose from 0.3 g to 2.75 g L^−1^ produced an improvement in the adsorption % of Pb (II) ions from 22.3 to 85%, respectively. This effect is credited to the increased available surface area and adsorption sites for the taking of Pb (II) ions.

#### Effect of foreign ions

Natural water may contain different anions and cations that probably influence the adsorption on the AC. Therefore, the adsorption % of Pb (II) by C_St20_ was assessed in the presence of certain potential ions like F^-^, Cl^-^, oxalate, acetate, Na^+^, and K^+^ (Table 6). The obtained results indicate that these ions did not significantly affect the adsorption of Pb (II). It was observed that only Ca^2+^ shows a negative effect on the adsorption process at higher concentrations (200 ppm), whereas, for low concentrations of Ca^2+^ (10 ppm), the adsorption % reached 97.85%.

**Table 6:** Effect of potential foreign ions on the adsorption of Pb(II) by C_St20_.

Foreign ions (200 ppm)	%Adsorption
F^−^	84.10
Cl^−^	100
Oxalate	65.99
Acetate	100
K^+^	92.60
Na^+^	97.98

#### Pb (II) adsorption isotherm modeling

The equilibrium data for Pb (II) adsorption by C_St20_ has been modeled using Langmuir, Freundlich, and Dubinin–Radushkevich isotherms. The obtained results for the three models are displayed in Table 7. The obtained results can be summarized in the following points:

1. Langmuir parameter (b) for Pb (II) (0.037) indicated favorable adsorption of Pb (II) on AC.
2. The Freundlich parameter (n) is greater than 1, indicating favorable adsorption of Pb (II) on AC.
3. Both Freundlich and Langmuir models are appropriate for expressing the adsorption of Pb (II) on the AC, as indicated by the high values of correlation coefficient (R^2^) (0.977 and 0.934, respectively). The model that better fits the adsorption of Pb (II) on C_St20_ is the Langmuir adsorption model, as revealed by the higher R^2^.
4. The free energy calculated from Dubinin–Radushkevich isotherm is less than 8.0 kJ mol^−1^, indicating that the adsorption of Pb (II) is mostly a physisorption process.

**Table 7:** Langmuir, Freundlich, and Dubinin-Radushkevich (D-R) isotherms constants and Kinetic parameters for adsorption of Pb (II) by C_st20_.

Isotherms model	Constants		Kinetic models	Constants	
Langmuir	q_m_ (mg g^−1^)	130.89	First-order kinetic Eqs	q_1_e (mg g^−1^)	50.96
	b (L mg^−1^)	0.037		K_1_ (min)^−1^ .10^3^)	3.638
	R^2^	0.999		R_1_^2^	0.544
Freundlich	K	5.367	Second-order kinetic Eqs	q_2_e (mg g^−1^)	41.084
	n	1.445		K_2_ [(g mg^−1^ min^−1^).10^4^]	29.038
	R^2^	0.934		R_2_^2^	0.999
Dubinin-Radushkevich (D-R)	β (mol^2^ kJ^−2^)	8.17 × 10^–7^	Intraparticle diffusion Eqs	K_int_ (mg g^−1^ min^−1/2^)	1.265
	E (kJ mol^−1^)	0.782		C (mg g^−1^)	20.764
	q_m_ (mg g^−1^)	61.460		R^2^_int_	0.601
	R^2^	0.545	Boyd Eqs	I	0.968
				R^2^	0.864

#### Pb (II) adsorption kinetic modeling and rate-controlling step

In order to describe the adsorption kinetics of Pb (II), the pseudo-first-, pseudo-second-order, intraparticle diffusion, and Boyd models were utilized. The obtained results are presented in Table 7. The obtained R^2^ value for the pseudo-first-order model is obviously lower than that of the pseudo-second-order model (0.544 and 0.999, respectively), suggesting that the pseudo-second-order kinetics model is appropriate to describe the Pb (II) removal.

The intraparticle diffusion model was utilized to study the mechanism of mass transport and to indicate the rate-limiting step during Pb (II) adsorption on the surface of C_St20_. The obtained parameters are presented in Table 7. Since the intercept of the intraparticle diffusion curve is not equal to 0 (= 20.764), it is suggested that intraparticle diffusion is not the only step that controls the adsorption process, and there is another step allying with it.

Further, the Boyd kinetic model has been taken into consideration; the calculated results are presented in Table 7. The obtained data indicated that the intercept is not equal to 0 (= 0.968), which suggested that the adsorption is also governed by film diffusion^48^.

#### Mechanism of Pb (II) adsorption by the AC

Building on the obtained experiments and results, the underlying mechanisms of Pb (II) adsorption by AC are suggested to be physisorption processes, including electrostatic interaction, beside ion exchange (Fig. 11). The electrostatic attraction takes place between the positively charged lead ions and the negatively charged surface function groups of AC such as –O^−^. Besides, the ion exchange of Pb (II) and protons from AC surface functions groups according to the following equation^60^:

$$\left( {{\text{AC}} - {\text{OH}}} \right)_{{2}} + {\text{ Pb }}\left( {{\text{II}}} \right) \to \left( {{\text{AC}} - {\text{O}}} \right)_{{2}} {\text{Pb }} + {\text{ 2H}}^{ + }$$

The synergetic combination of these mechanisms is responsible for the efficient adsorption of Pb (II) from water by the prepared agro-based AC. Furthermore, pore filling is a likely mechanism that contributes to the removal of Pb (II) from the aqueous medium, where Pb (II) penetrates the mesopores and micropores of AC that offers an extra capacity for uptake of Pb (II). Both intraparticle and film diffusion control the adsorption process^60^.

### Real sample applications

Markedly, compared to other physiochemical treatment procedures, adsorption is competent in the elimination of different heavy metals from aqueous solutions^35,49,61^. For instance, mesoporous silica based adsorbents with different anchored ligands have been employed for the removal of various metals from contaminated water. Ni (II) has been eliminated from waste water by silica based composite adsorbents^61^. As well, Cd (II) has been successfully eliminated from contaminated water by 2,2′-biquinoline-4,4′-dicarboxylic acid-embedded mesoporous silica^62^. Similar approaches has been applied for recovery of Sm (III) utilizing 4-chlro-2-mercaptophenyl)carbamodithioate immobilization onto mesoporous silica^63^ and Pb (II) using 2,4,6-trichlorophenol-anchored mesoporous silica^64^. Rare-earth Yb(III) ions have been recovered from aqueous solution by 1E,1`E,1``E,1```E (tetrakis(3-carboxysalicylidene)) naphthalene-1,2,5,5-tetramine-bound mesoporous silica monoliths^65^. However, mesoporous silica exhibit some limitations as an adsorbent such as difficulty of synthesis, distributed particle size, and unstability of its aquesous suspension. Thus, it is needed to develop better adsorbing materials. In this context, activated carbon shows many merits such as cost-efficiency, simple preparation, durability, well-defined porous structure, and fast adsorption and desorption.

Lead may occur in drinking water because of the corrosion of lead-containing plumbing materials and pipes. By virtue of its high toxicity, harmful impact on human health, serious behavioral and physical influences on children and fetuses, and persistence in the human body, the United States Environmental Protection Agency (USEPA) settled the maximum allowed level of lead in drinking water to be zero. The USEPA assessed that about 20% of the individual’s exposure to lead is via drinking water. The USEPA regulations recommend continuous monitoring of the level of lead in water systems and suggest additional action if lead is detected in water. These facts imply the need for new, effective technology for removal of lead from water^66,67^.

The applicability of the prepared activated carbon C_St20_ for adsorption of Pb (II) from discrete water samples, e.g., distilled water, tap water, wastewater, and underground water, was investigated utilizing three spiked concentrations (5, 10, and 15 ppm). The tests were done using 25 mL of filtered water samples after adapting their pH to 4.5, adding 0.05 g of the C_St20_ sample, and stirring the mixture. The obtained results (Table 8) show that the recovery was excellent, ranging from 95.12 to 99.23%, with a relative standard deviation (RSD) ≤ 3.0. The results obtained reveal the excellent performance of the prepared agro-waste-derived AC for adsorption of Pb (II) and efficient water remediation.

**Table 8:** Recovery of Pb(II) from different water samples using C_St20_ activated carbon (*n* = *5*).

Water samples	Conc. added, ppm	Recovery, %	RSD, %
Distilled water	5.0	99.00	0.470
	10.0	97.70	0.234
	15.0	96.21	0.543
Tap water	5.0	98.79	0.344
	10.0	97.60	0.372
	15.0	95.67	0.563
Wastewater	5.0	99.23	0.763
	10.0	96.86	0.326
	15.0	95.54	0.436
Underground water	5.0	98.82	0.634
	10.0	95.96	0.574
	15.0	95.12	0.534

### Regeneration and reuse of the prepared AC

**Figure 14:**
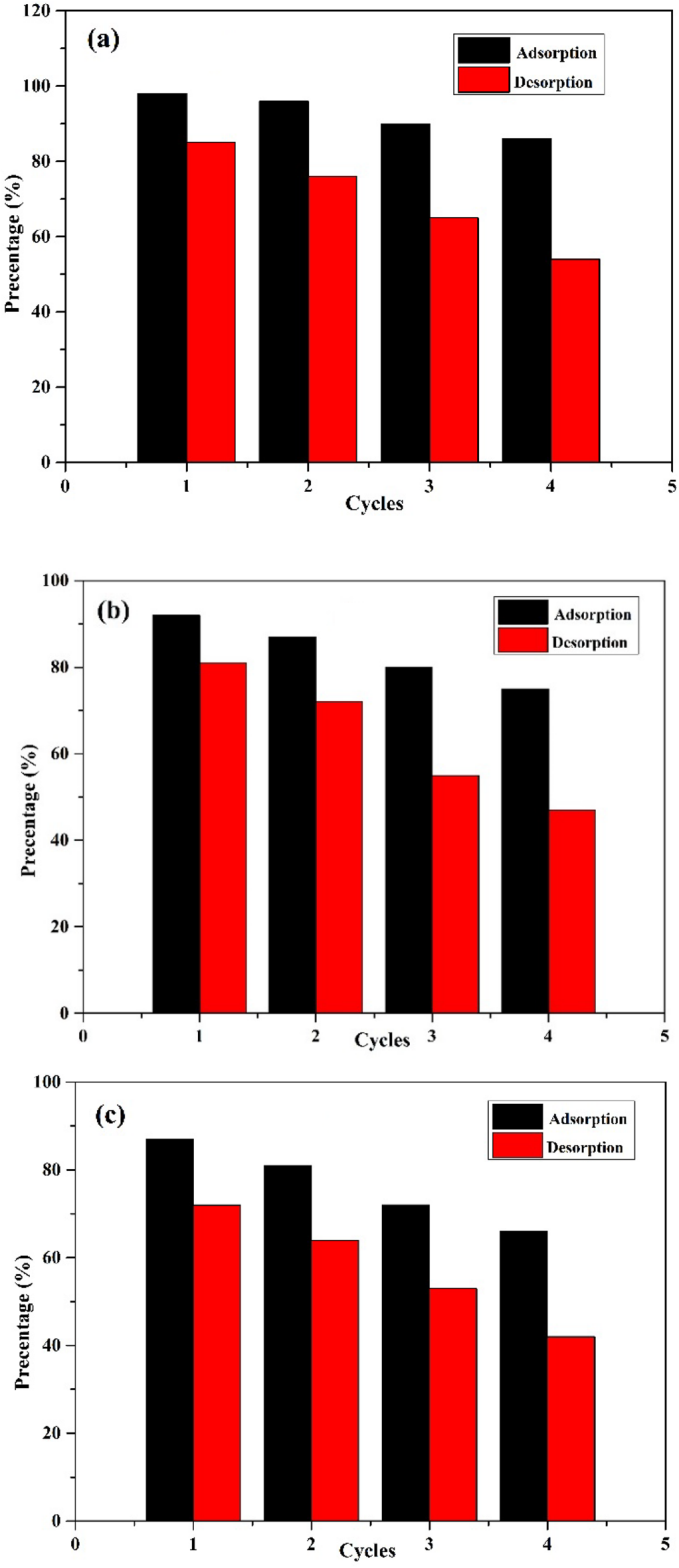
Adsorption/desorption percentages cycles of (**a**) C_zn21_, (**b**) C_Na13_, and (**c**) C_st20_.

The prepared AC was regenerated utilizing NaCl as the desorbing agent and reused for adsorption (Fig. 14). Following four adsorption/desorption cycles, the adsorption efficiency was found to decrease from 98 to 86% for C_zn21_, from 92 to 75% for C_Na13_, and from 87 to 66% for C_st20_ at an initial pollutant concentration of 50 ppm at room temperature for 120 min of contact time. It was deduced that increasing the adsorbent regeneration cycles led to a decrease in both the adsorption and the desorption effectiveness (Fig. 14). These results indicated the feasibility of the adsorbent regeneration in multiple successive pollutant adsorption–desorption cycles using salt without the application of any organic solvent. The regenerated low-cost materials could be reused, and they have potential applications for intended purposes in water treatment. The developed approach corroborates the eco-design concept that aims to minimize the eco-impact of a product via integrating the environmental aspects into the product development process. Herein, the life cycle of peanut shells involves their conversion into a useful product that can be repeatedly utilized for water purification and removal of dye/metal pollutants^68^.

### Comparison of the developed agro-based AC adsorbent and existing adsorbents for MB and Pb (II)

Table 9 compared the developed peanut shells-derived activated carbon as an adsorbent for the removal of MB and Pb (II) and other reported adsorbent materials with respect to adsorption capacity, removal %, and conditions of adsorption^64,69–75^. Only cross-linked chitosan-zeolite composite^69^ has better adsorption capacity than the prepared AC; however, it requires a contact time of 24 h to achieve this adsorption capacity, while the developed AC accomplished 130.89 mg g^−1^ adsorption capacity within 50 min. Furthermore, its synthesis process is complicated, and it is not as environmentally friendly as the suggested AC. Besides, the AC fibers modified by L-cysteine^70^ have similar adsorption capacity to the prepared AC for Pb (II) adsorption, yet it needs seven times longer contact time (6 h), it achieved much lower removal % of Pb (II) (51.55%), and its synthesis is more complicated compared to the suggested AC. All other investigated adsorbents exhibit poorer adsorption capacity while requiring longer contact time. It is notable that most of the reported adsorbent work at pH ranged from 3.5 to 5.5^69–73^, except dolomite-quartz@Fe_3_O_4_^74^ and AC nanoparticle impregnated on lightweight expanded clay aggregate^75^ that require a basic pH of 8.5 and 6.0, respectively.

**Table 9:** An overview of the adsorbents and conditions used for the adsorption of lead and MB in the literature.

Adsorbate	Adsorbent	Conditions of adsorption	Adsorption capacity	% Removal	Application	Ref No.
Pb (II)	Dolomite-quartz@Fe_3_O_4_	pH 8.5, 25 °C, contact time = 140 min	71.68 mg g^−1^	95.02%	Simulated aqueous solutions	^74^
Pb (II)	Cross-linked chitosan-zeolite composite	pH 4.5, 24 h	275 mg g^−1^	˃ 90%	Simulated aqueous solutions	^69^
Pb (II)	AC nanoparticle impregnated on lightweight expanded clay aggregate	pH 6, contact time = 180 min at 298.25 K	22.83 mg g^−1^	98.845%	Simulated aqueous solutions	^75^
Pb (II)	AC Prepared from Juniperus procera Leaves	pH 4.6, contact time = 100 min	–	98%	Simulated aqueous solutions	^71^
Pb (II)	Polypyrrole-Based AC	pH 5.5, contact time = 4 h	50 mg g^−1^	85%	Simulated aqueous solutions	^72^
Pb (II)	Commercial AC	pH 5.5–6	9.30 mg g^−1^	–	Simulated aqueous solutions	^73^
Pb (II)	AC fibers modified by L-cysteine	pH = 5.5, contact time = 6 h, temperature = 303 K	179.53 mg g^−1^	51.55%	Simulated aqueous solutions	^70^
Pb (II)	2,4,6-trichlorophenol (TCP) ligand immobilized onto mesoporous silica	pH 3.5, contact time = 60 min, room temperature	172.87 mg g^−1^	99.98%	Simulated aqueous solutions	^64^
Pb (II)	AC from peanut shells	pH 4.5, contact time = 50 min	130.89	˃ 95%	Tap, waste, and underground water	This work
MB	Commercial AC modified with sodium dodecyl sulfate	pH 5.0, contact time = 120 min	232.5 mg·g^−1^	≈95%	Tap, raw, and wastewater	^77^
MB	Coal-based AC	pH ˃ 6.0, contact time ˃ 8 h	15.5 mg·g^−1^	90%	Simulated aqueous solutions	^78^
MB	AC derived from lignocellulosic agriculture wastes	pH 4.0, contact time = 30 min	148.8 mg·g^−1^	69%	Simulated aqueous solutions	^79^
MB	AC derived from Rumex abyssinicus plant	pH 9, contact time = 60 min	322 mg·g^−1^	99.9%	Simulated aqueous solutions	^80^
MB	AC derived from Capsicum Straw	pH 5.0, contact time = 200 min	34.12 mg·g^−1^	–	Simulated aqueous solutions	^76^
MB	AC from peanut shells	pH 10, contact time = 50 min	303.03	˃ 95%	Simulated aqueous solutions	This work

Regarding MB, only AC derived from *Rumex abyssinicus* plant^76^ showed similar adsorption capacity under adsorption conditions like the developed AC. All other investigated adsorbents have lower adsorption capacities^77–80^. Additionally, most of the reported literature requires longer contact time^76–78,80^.

These results suggest that the peanut shells sourced ACs are a very promising adsorbent for the purification of contaminated water, and it has a great potential to participate in achieving the 6th goal of the United Nations sustainable development goals “Clean Water and Sanitation”^81^.

## Conclusions and future aspects

In this study, AC adsorbent was prepared from peanut shells and structurally confirmed by FTIR and SEM analysis. Both MB dye and Pb (II) have been adsorbed significantly from aqueous solutions on the AC sourced from peanut shells as a cost-efficient, green, and renewable carbon source. The highest adsorption of MB was achieved at basic pH within 1 h, while for Pb (II), the adsorption capacity was maximum at pH 4.5 within 1 h. The kinetics of MB adsorption obeyed the pseudo-second-order model, and the Langmuir adsorption model is the best-fit model for the adsorption isotherm of MB on all AC samples. The adsorption mechanisms involved in MB-AC interaction include electrostatic interaction, π–π interaction, and hydrogen bonding, while Pb (II) is anticipated to interact with AC via electrostatic interaction and ion exchange.

As per the correlation coefficient, the obtained kinetic results were best fit by the Langmuir isotherm with maximum adsorption capacity of 303.03 mg g^−1^ for MB and 130.89 mg g^−1^ for Pb (II). The AC successfully removed MB and Pb (II) from aqueous solutions with %removal exceeding 95%. Thus, AC has been applied for remediation of waste and groundwater with excellent %removal ranging from 95.12 to 99.0%. The removal efficiency revealed a potential application of the peanut shells-derived AC material for the elimination of dye and heavy metal from wastewater, which will be highly economical and convenient. The prepared AC showed adsorption capacity greater than many reported adsorbing materials within a shorter contact time. The prepared material is promising for future applications as an adsorbent for the removal of other dyes and heavy metals from industrial wastewater.

### Supplementary Information

Supplementary Figure S1.

## Data Availability

Data is provided within the manuscript or supplementary information files.
