# Supplementary material for: Inclusive study of peanut shells derived activated carbon as an adsorbent for removal of lead and methylene blue from water
Source: Sci Rep. 2024 Jun 12;14:13515. doi: 10.1038/s41598-024-63585-9 (PMC11169236; doi:10.1038/s41598-024-63585-9)
Supplement: Supplementary file 1 — Supplementary Figure S1. [file 41598_2024_63585_MOESM1_ESM.pdf]

Scientific Reports

Supplementary Materials

Inclusive study of peanut shells derived activated carbon as an adsorbent for removal of lead and methylene blue from water

Heba M. Hashem, Mahmoud El-Maghrabey, Rania El-Shaheny*

Department of Pharmaceutical Analytical Chemistry, Faculty of Pharmacy, Mansoura University, Mansoura 35516, Egypt

*Corresponding author: R. El-Shaheny, email: rania_yomna@mans.edu.eg, Phone: 0201068744339, Fax: 050/ 2247496.

Orcid:
HMH: 0000-0002-6156-3582
MHE: 0000-0003-4661-6612
RNE: 0000-0002-8830-4222

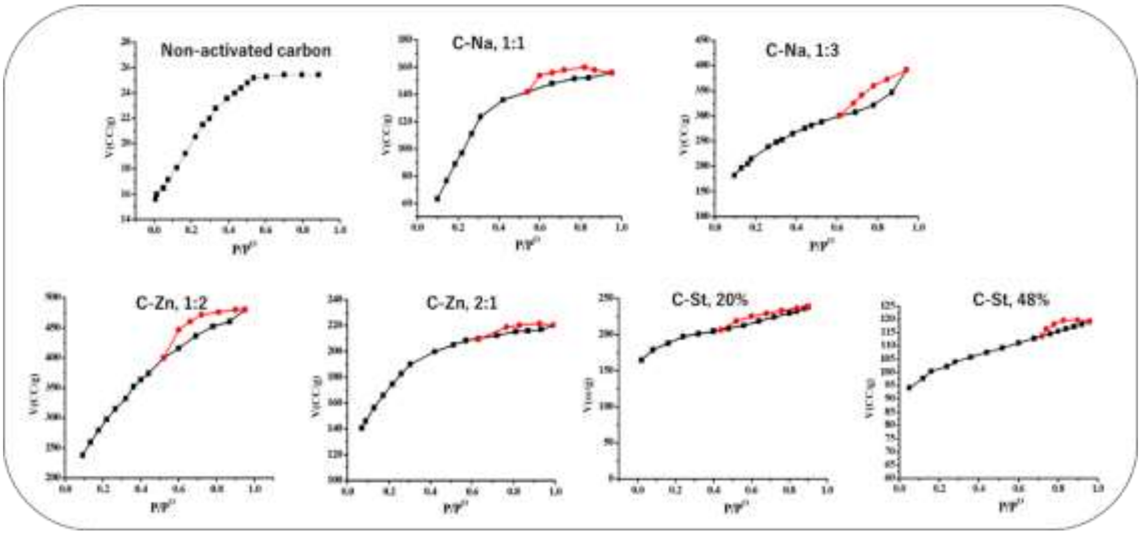

Figure S1. Nitrogen adsorption-desorption isotherms at-196°C for the non-activated carbon and ACs.
